# A Narrative Review of Cell-Based Approaches for Cranial Bone Regeneration

**DOI:** 10.3390/pharmaceutics14010132

**Published:** 2022-01-05

**Authors:** Maria I. Falguera Uceda, Silvia Sánchez-Casanova, Clara Escudero-Duch, Nuria Vilaboa

**Affiliations:** 1 Hospital Universitario La Paz-IdiPAZ, Paseo de la Castellana 261, 28046 Madrid, Spain; mfalguer@msn.com (M.I.F.U.); silvia.scasanova@gmail.com (S.S.-C.); claraescuderoduch@gmail.com (C.E.-D.); 2 Unidad de Gestión Clínica de Cirugía Maxilofacial Hospital Universitario Reina Sofía, Avenida Menéndez Pidal s/n, 14004 Córdoba, Spain; 3 Centro de Investigación Biomédica en Red de Bioingenieria, Biomateriales y Nanomedicina, CIBER-BBN, 28046 Madrid, Spain

**Keywords:** cranial bone, biomaterials, cell therapy, gene therapy

## Abstract

Current cranial repair techniques combine the use of autologous bone grafts and biomaterials. In addition to their association with harvesting morbidity, autografts are often limited by insufficient quantity of bone stock. Biomaterials lead to better outcomes, but their effectiveness is often compromised by the unpredictable lack of integration and structural failure. Bone tissue engineering offers the promising alternative of generating constructs composed of instructive biomaterials including cells or cell-secreted products, which could enhance the outcome of reconstructive treatments. This review focuses on cell-based approaches with potential to regenerate calvarial bone defects, including human studies and preclinical research. Further, we discuss strategies to deliver extracellular matrix, conditioned media and extracellular vesicles derived from cell cultures. Recent advances in 3D printing and bioprinting techniques that appear to be promising for cranial reconstruction are also discussed. Finally, we review cell-based gene therapy approaches, covering both unregulated and regulated gene switches that can create spatiotemporal patterns of transgenic therapeutic molecules. In summary, this review provides an overview of the current developments in cell-based strategies with potential to enhance the surgical armamentarium for regenerating cranial vault defects.

## 1. Introduction

The craniofacial region is an anatomically complex set of bone, cartilage, blood vessels, soft tissues and nerves. Compared with the endochondral ossification of the major part of human skeleton, craniofacial bones, derived from the cranial neural crest and paraxial mesoderm [1], display a more complex ossification pattern, in which cranial vault is formed by intramembranous ossification, whereas the cranial base is formed endochondrally. Reconstruction of cranial vault defects caused by trauma, surgical procedures or congenital defects has historically been performed by replacing the damaged tissue with various materials. The first surgical approaches to the restoration of the cranial vault date back to the seventeenth century, with the implantation of dog cadaver bone in a cranial defect. Subsequently, the use of a variety of materials was described, many of which were discarded due to their cost, lack of stability or adverse biological reactions, among other reasons [2]. The relevance of a successful cranioplasty lies in its protection of the intracranial content, the aesthetic rehabilitation with the psychosocial implications around this aspect, and its control of alterations in the cerebrospinal fluid and blood flow. The control of such alterations can prevent neurological complications associated with craniectomies, such as syndrome of the trephined, which has an overall incidence estimated between 2% and 24% [3]. The rate of cranioplasties performed in recent years has progressively increased, reaching an estimated incidence of 0.72 per 100,000/year in the United Kingdom [4] or 3.62 per 100,000/year in South Korea [5]. Infection is one of the most common and most serious complications of cranioplasties, given that it can cause neurological and systemic complications and generally requires the removal of the prostheses.

**Figure 1:**
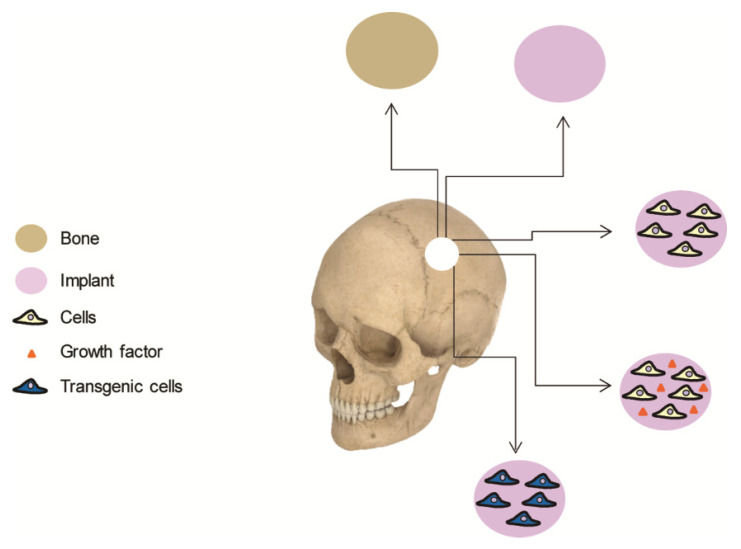
Current clinical methods and experimental cell-based approaches for cranial bone repair. The figure includes an image retrieved from https://free3d.com/es/modelo-3d/caucasoid-male-skull-4837.html, accessed on 30 August 2021.

Bone autograft remains the safest and most effective procedure for cranioplasties in most of the cases. Given that autografts suffer from same drawbacks, the scientific community has long been searching for biomaterials to replace the lost bone. These materials are designed to mimic both the composition and structure of bone, although no single biomaterial has yet been discovered that possesses the biological properties of autologous bone. By implanting osteogenic cells on supportive biomaterials, bone tissue engineering offers an attractive alternative that enhances natural bone regeneration (Figure 1). This article aims to review cell-based therapies for calvarial bone repair and is divided into the following sections: (i) an overview of the current clinical approaches used in cranioplasty, including brief comments on their limitations; (ii) a review of cell-based strategies for calvarial tissue engineering, including clinical trials and preclinical research; (iii) a summary of cell-based gene therapy approaches, covering both unregulated and regulated gene switches that can create spatiotemporal patterns of regenerative molecules; and (iv) some possible future directions for this rapidly growing area.

A search of the literature was conducted until 15 October 2021. The eligibility criteria were as follows: (a) original articles reporting preclinical and clinical studies; (b) systematic and narrative reviews. Letters to editors, commentaries and conferences abstracts were excluded. PubMed and Web of Science online databases were searched using a combination of the terms: “cranioplasty”, “calvaria”, “skull”, “cranial”, “biomaterial”, “implant”, “scaffold”, “cell therapy”, “bone tissue engineering”, “3D printing”, “bioprinting”, “extracellular vesicles”, “extracellular matrix”, “conditioned media”, “gene therapy”, “transgene” and “growth factor”. A screening of the full texts of the retrieved articles was performed next to select relevant original articles and reviews addressing calvarial bone repair. Retrieved studies that were not directly related to calvaria bones were excluded. Finally, 196 studies published between 1985 and 2021 were considered by the authors as relevant for the topic and included in this review.

## 2. Clinical Approaches for Cranial Bone Repair

### 2.1. Bone Grafting

A critical size cranial defect is that lacking enough bone tissue to heal spontaneously. The size of a defect to be considered critical depends on various factors that affect bone healing, such as the location of the lesion, patient age and the condition of the surrounding bone and soft tissues in terms of infection, previous radiotherapy or diseases affecting bone metabolism, such as diabetes or osteoporosis. Currently, autologous bone transplantation is the gold standard of cranial reconstruction surgery. Autologous bone grafts do not cause host rejection and they integrate well with the cranial cavity, resulting in a lower risk of fracture [6]. Autologous bone grafting is widely accepted due to its low cost and availability, in addition to the perception of a better result from the patient. However, high failure rates due to infections and graft resorption have been reported, ranging from 14% to 50% [7,8]. The ideal option after a craniectomy is immediate replacement of the removed cranial bone fragment whenever possible. In the absence of infectious foci, with adequate rigid osteosynthesis and soft tissue coverage, this procedure leads to proper integration with the adjacent bone. Bone graft incorporation occurs by osteoconduction, which depends on the healthy bony edges of the defect being in stable contact with the graft that is replaced by new bone formation. In cases where the bone fragment is not viable, e.g., due to tumor involvement or comminuted fracture, immediate replacement is not possible and a bone graft harvested from another area can be used to cover the defect with a good integration rate. The parietal bone is often preferred for obtaining this graft [9], because it is a membranous bone that maintains bony volume, especially if it is rigidly fixed. In addition to being easily accessible, cranial bone has the advantage of having 2 diploes that can be separated, using the outer diploe to correct the skull defect and replacing the inner diploe in the donor area [10]. Parietal autografts have a low rate of resorption, which has been attributed to the delayed revascularization that characterizes cortical bone, resulting in enhanced volume maintenance [11]. However, the available tissue is often insufficient to cover the defect. Moreover, the diploic space of children younger than 4–5 years of age is absent or underdeveloped. As an alternative, bone from the ribs and the iliac crest has been employed for cranial reconstruction. However, bone from these sources is more difficult to adapt to the cranial convexity and can leave contour irregularities; thus, it is recommended only as a second option, and in areas covered by hair. Rib and iliac crest grafts are not first choices in pediatric patients due to the risk of chest wall deformity and potential interference with gait stability, respectively. Due to the osteogenic ability of underlying dura during childhood, particulate bone grafts, harvested either from the ectocortex or the endocortex of a full thickness bone segment of the skull, are an excellent option for the pediatric population [12]. Moreover, a developed diploic space is not required for harvesting particulate bone grafts. However, particulate grafts lack structural integrity until healing nears completion and they cannot be used in cranial sites without underlying bony or dural support.

In the case of decompressive craniectomies, in which the cranial defect is reconstructed only after resolution of the cerebral edema, subcutaneous or extracorporeal preservation of the patient’s bone allows further reimplantation [13,14]. A classic preservation method places the explanted bones in a subcutaneous pocket at the abdominal wall, until the cranioplasty is performed. Alternatively, to avoid added morbidity of a second surgical site and patient discomfort, craniectomy bone flaps can be frozen until the time that the cranioplasty is performed. Cryopreservation is widely accepted due to its low cost and the simplicity of its replacement, although numerous studies report high failure rates due to infections or graft resorption, especially in younger patients, large defects or long storage time before replacement [8,15]. Sterilization of the cryopreserved bone flap, e.g., by autoclaving or ethylene oxide treatment, before reimplantation, reduces the risk of infection but can result in increased risk of bone flap resorption [16,17].

The use of cadaveric cranial grafts in cranioplasty was associated with complications secondary to infection and bone resorption. Therefore, the use of allografts in contemporary cranial reconstruction remains relatively rare [18,19]. However, human demineralized bone matrix obtained from the processing of bone allografts has been successfully employed, alone or in combination with autologous bone chips, for cranial reconstruction [20,21].

### 2.2. Biomaterials

As mentioned earlier, autologous bone transplantation is the gold standard of craniofacial skeleton reconstruction surgery. Although this method is suitable for small- to medium-sized defects, problems associated with morbidity of the donor site and tissue availability hamper its use in reconstructive treatments of large defects. In addition, the risk of bone resorption with an autologous implant remains high in children and adolescents when compared with adults [15,22,23]. Therefore, the use of autologous bone for the reconstruction of the cranial vault currently coexists with the use of biomaterials. Biomaterials are used for cases in which there is not enough bone stock available or its use is not recommended, with varying indications according to clinical criteria, personal preference of the surgeon and cost. Biomaterials have made it possible to restore large cranial defects with acceptable rates of complications, avoiding the need to deplete a tissue donor site from the patient and the complications associated with bone grafts, such as resorption, lack of osseointegration and infections. An ideal biomaterial for pediatric cranioplasty should not only integrate with the adjacent bone but also adapt to its developing anatomy and dynamic growth; i.e., it should interact with the child’s skull. To stress the importance of the interactions between biomaterials and tissues, the Consensus Conference on Definitions of Biomaterials for the Twenty-First Century held in 2018 defined a biomaterial as “a material designed to take a form that can direct, through interactions with living systems, the course of any therapeutic or diagnostic procedure” [24].

The ideal biomaterial for cranioplasty should be biocompatible; radiolucent; non-toxic; with low complication rates; easy to use in the operating room; suitable for bending and molding, thus allowing the preparation of patient-specific implants; with excellent cosmetic results and low cost. The only material that might meet all these specifications is autologous bone, whereas the ideal biomaterial that fulfills all these requirements remains to be discovered [25]. Biomaterials for bone substitutes can have a natural or synthetic origin, meeting the requirements of the patient’s needs to a greater or lesser extent [7,26]. Some commonly used materials in adult cranioplasty are problematic for applications in pediatric cranioplasty because they lack adapting capacity. Rigid biomaterials cannot adapt to a growing skull, which can result in intracranial migration of the implant or asymmetry of cranial growth and deformity. Unfortunately, research on the effects of biomaterials for cranioplasty of large defects on human skull development is scarce. It has been hypothesized that after 5 years of age, when approximately 90% of cranial growth has been achieved, rigid biomaterials can be used for reconstruction with minimal or no risk of intracranial migration or adverse effects on cranial growth [27]. The most common biomaterials in cranioplasties include materials of synthetic origin, such as titanium, polymethylmethacrylate (PMMA), polyetheretherketone (PEEK) and bioceramics such as hydroxyapatite (HA) or beta tricalcium phosphate (β-TCP) [25,28].

Titanium is biocompatible, chemically stable and suitable for the manufacture of patient-specific implants. Compared with other biomaterials used for cranioplasty, reconstruction using titanium provides for immediate and durable skull protection [29]. Titanium tightly binds to adjacent bone, in a process known as osseointegration, in which living bone lies in direct apposition to the metal surface without intervention of a fibrous tissue interface. Cranioplasty procedures performed with titanium result in overall complication rates of up to 30%, but lower infection rates than autologous bone, PMMA or ceramic materials [7,26,30]. Some disadvantages are that titanium is expensive, with high thermal conduction and is radiopaque [31]. Titanium cranioplasties can be performed using either curved meshes that are adapted in situ to the individual patient or custom-made prostheses that are prepared using computer-aided design. A comparative study showed that custom-made cranioplasties resulted in shorter and easier operations than curved meshes and provided for complete coverage of large defects with better cosmetic outcomes [32]. Curved meshes, available for emergency reconstruction, only provided satisfactory esthetic results in small defects. Therefore, titanium custom-made implants are recommended for children older than 5 years and for adult patients with large cranial defects, e.g., those resulting from decompressive craniotomy, in which donor site morbidity precludes the use of autologous bone [27].

Synthetic polymer-based bone substitutes, a heterogeneous group of materials, include PMMA, polylactic acid (PLA), poly(ε-caprolactone) (PCL), polyethylene (PE) and PEEK. PMMA is an acrylic polymer that is formed by mixing a liquid methyl methacrylate monomer and a powder containing methyl methacrylate-styrene co-polymer or pre-polymerized PMMA. The powder contains an initiator, di-benzoyl peroxide, and N,N-dimethyl-p-toluidine is added to activate polymerization at room temperature. When the components are mixed, the liquid monomer polymerizes around the co-polymer or pre-polymerized PMMA powder particles to form hardened PMMA. Hydroquinone is also added to prevent premature polymerization due to light or high temperature exposure. Polymerization of PMMA occurs as an exothermic reaction, generating a paste that the surgeon can easily model and that slowly cools down and hardens into a solid within a few minutes. The result is a stable and strong material with compression and stress resistance higher than those shown by HA. In addition to being moldable, PMMA is cheap and radiolucent. Given that PMMA is brittle, it is often used in combination with titanium. Titanium meshes are often used to shield the PMMA from venous pulsation during the curing period as well as to prevent thermal injury to the underlying brain tissue. In general, PMMA is a safe biomaterial, with complication rates of 22.7%, similar to other materials used in cranioplasties [7]. As for disadvantages, it does not osseointegrate, and it has infection rate of 7.8%, higher than those reported for other materials [33]. Lastly, incomplete polymerization of PMMA is of high concern because it can result in monomers with neurotoxic effects [34]. The exothermic reaction that occurs during polymerization can be harmful to the brain; therefore, in situ polymerization of PMMA is not recommended for pediatric patients. Custom-made PMMA implants have been successfully used in children’s cranioplasties by preventing exposure of the brain tissue to high temperatures during intraoperative molding and the neurotoxicity derived from monomer residues [35].

Due to their similarity to the mineral phase of bone, bioceramics, and among them, calcium orthophosphates such as HA, have been extensively used to prepare implants for cranioplasty [28]. HA obtained by means of sintering at high temperatures is too brittle, poorly bioactive and, due to its stoichiometry and high crystallinity, non-biodegradable [36]. Adjustment of the porosity of HA implants has been successfully addressed as a strategy to enhance osteoconductivity and biological performance. A study conducted with 2697 patients who underwent cranioplasty with custom-made, sintered, porous HA implants revealed a low incidence of adverse events (5.72%) [37]. These implants are also a good option for pediatric patients, although complications after HA cranioplasty occurred in 20.8% of children younger than 7 years of age compared with 3.8% of adults and children older than 7 years [37,38]. Granular β-TCP, a much more soluble ceramic than HA, has also been used in cranial repair in combination with supporting meshes [39,40]. Calcium phosphate cements (CPCs) are moldable systems that combine a calcium phosphate-based powder and an aqueous solution, which upon mixing hardens in situ into a crystalline solid. Unlike the acrylic cement PMMA, the setting reaction of CPC is not exothermic, adding safety and allowing the incorporation of thermolabile drugs or biologically active molecules, e.g., antibiotics [41]. However, a meta-analysis study revealed a mean complication rate of 13% in cranioplasties conducted with CPCs [42]. The risk of complications remained several years after the procedure, resulting in implant failure that required a second surgery. As with HA, CPCs are fragile materials that are better suited for cranioplasties of small defects. In the presence of large defects that require mechanical strength, CPCs are used in combination with a titanium mesh [41].

PLA and PCL are biodegradable polyesters that currently have a role in pediatric skull surgery as resorbable fixation plates that are replaced by bone during its natural degradation. They are highly biocompatible, with a slow degradation rate that preserves their mechanical features [43]. Their usefulness as materials for preparing custom-made cranial implants is described in some studies, but they have not been extensively clinically employed. Demineralized bone matrix has been combined with bioresorbable mesh prepared with PLA. A construct of polymer is placed on the dura to provide for protective reconstruction, layered with demineralized bone matrix, with or without particulate bone graft, and then covered with an outer layer of polymer [44]. PCL has been used to prepare porous implants to cover burr-holes created to evacuate chronic subdural hematoma [45]. The implants were well tolerated, showing signs of osteointegration into the surrounding calvarial bone and yielding good cosmetic results.

**Figure 2:**
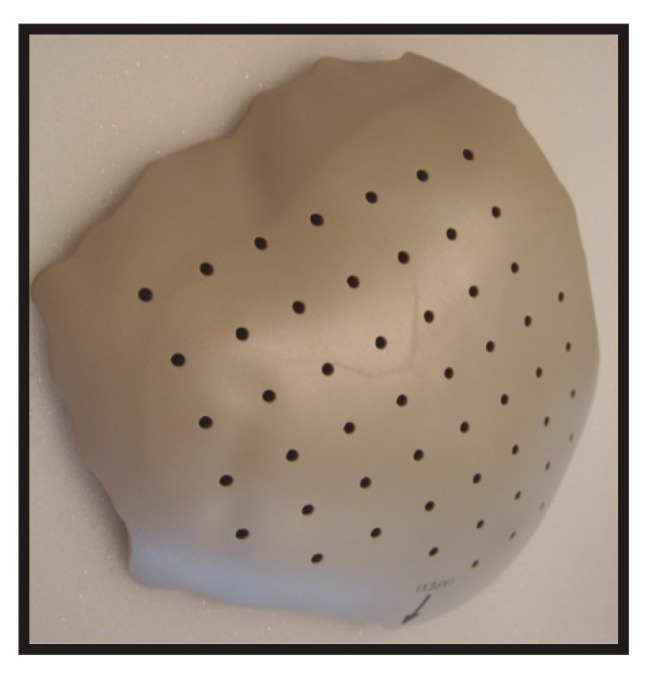
Patient-specific PEEK cranioplasty implant fabricated using computer-aided design and computer-aided manufacturing.

The most popular plastic cranioplasty materials are PE and PEEK. Porous PE implants can be custom-tailored to meet patient specifications. The main disadvantage of these implants relates to their limited ability to support osteoconduction [46], being used in pediatric patients as a temporary measure until skull growth and development of a robust diploic space allows a definitive cranioplasty using autologous bone graft [47]. PEEK is a semicrystalline thermoplastic aromatic polymer that can be sterilized without deformation using various methods (Figure 2). Its advantages include favorable mechanical properties, such as high strength and stiffness, with an elastic modulus comparable to that of human bone. It is radiolucent and can be prefabricated as a patient-specific implant [48,49]. However, PEEK is a highly hydrophobic material and therefore does not osseointegrate because it lacks bioactive potential [50]. Overall complication rates of 17–21% were identified with its use, mainly related to plate exposure, infection and lack of integration [7,26,51]. In pediatric cranioplasties, implant failures can be minimized by placing the PEEK implant with a bone gap lower than 6 mm [51].

## 3. Cell-Based Therapies for Cranial Bone Regeneration

Current cranial repair techniques combine the use of autologous bone grafts and biomaterials. In addition to being associated with harvesting morbidity, autografts are often limited by insufficient quantity of bone stock, a problem especially relevant in pediatric patients undergoing reconstruction of large skull defects. Reported complication rates in large autologous cranioplasty series are as high as 40%, with a mean reoperation rate of 25% [23,52]. Although biomaterials lead to better outcomes, their effectiveness is often compromised by unpredictable lack of integration, infection or structural failure. Bone tissue engineering offers the promising alternative of generating constructs composed of biomaterials including cells and/or growth factors that could enhance the outcome of reconstructive treatments [53]. The biomaterial provides a suitable environment for cells as well as structural support at the site of the defect. The ideal biomaterial should be bioresorbable, with mechanical properties comparable to native bone tissue and a 3-dimensional, highly porous structure with favorable surface properties to facilitate cell colonization and survival [54].

### 3.1. Human Studies

The clinical applications of stem cells in bone reconstructive surgery have been limited thus far to small case series. The first case report, published in 2004 [55], related to a 7-year-old girl who underwent progressive and disseminated chronic infection after reimplantation of cryopreserved cranial bone fragments, resulting in an unstable skull with marked bony defects that covered a total area of about 120 cm^2^. The amount of autologous cancellous bone available from the iliac crest to reconstruct the calvarial bone was very limited. To enhance the regeneration process, autologous stem cells derived from fat were applied in the defect, along with the autologous bone graft. Simultaneously with bone harvesting from the iliac crest, gluteal fat was obtained and processed during the ongoing surgical procedure to isolate autologous adipose-derived stem cells. The milled autologous bone was applied in the defect along with the stem cells which were kept in place using fibrin glue, obtained also from the patient who was subjected to plasmapheresis 2 days prior to surgery. Mechanical fixation was achieved using macroporous sheets based on PLA that also acted as barriers. The procedure was safe, with no signs of neurological deficits. Three months after cranioplasty, computed tomography showed marked ossification in the defect areas. Although it cannot be determined whether the effect was due to the conventional bone grafting or to the combination with autologous adipose-derived stem cell transplantation, the results encouraged subsequent studies on cranial reconstruction using stem cells.

Thesleff et al. [39] published the results of a clinical trial with four patients with large calvarial defects of different etiologies who underwent cranioplasties in which autologous adipose stem cells were transplanted. Three weeks before the cranioplasty, subcutaneous abdominal fat was harvested and used to isolate and expand adipose stem cells using autologous serum. The cells were extensively characterized in terms of viability, expression of phenotypic markers and in vitro osteogenic differentiation potential. Two days before the cranioplasty, the cells were combined with β-TCP granules and cultured until the procedure was performed. In two patients, the cells-containing granules were directly laid on the dura and then covered by a titanium mesh or a molded, biodegradable poly(L-lactide-co-glycolide) mesh. In the other two patients, a bilaminate procedure was performed. First, a sheet of biodegradable mesh was placed on the dura and the cell-containing granules were applied to the mesh. A second layer of polymeric mesh was molded and placed on the outlay calvaria defects. The procedure was safe, without complications up to 3 months after cranioplasty, when increased ossification was quantified by means of computed tomography. Nevertheless, the 6-year follow-up results of these 4 cases, as well as of a fifth patient undergoing the same procedure, were unsatisfactory [40]. All patients except one had to be reoperated. Signs of resorption of the graft were observed in three cases out of five. After a follow-up period of 36 to 52 months, another study from the same group reported unsatisfactory results in five patients transplanted with β-TCP granules containing autologous adipose cells [56]. The three patients out of five who received a resorbable mesh sustained at least mild resorption of the adipose stem cell-seeded constructs. The two patients with satisfactory clinical and radiological ossification had either an inner or an outer mesh from titanium, suggesting that cranial mesh layers in this type of procedure should be made of rigid, non-resorbable material to sustain the dural pulsations to which these cranial wounds are exposed.

Due to impaired proliferation rates and senescence issues, it might be difficult to obtain large quantities of autologous stem cells from older patients [57,58]. A clinical trial with three patients explored the use of allogenic stem cells in cranioplasty [59]. Mesenchymal stem cells (MSCs) were obtained from the bone marrow of donors, expanded and cryopreserved until the procedure was scheduled. Nine days prior to surgery, the cryopreserved cells were thawed, cultured for approximately 1 week, combined with β-TCP granules and further incubated for 2 days. A bilaminate procedure using biodegradable meshes was performed as described earlier. Computed tomography performed at 3 and 6 months postoperatively revealed good restoration of the cranial defects. Thereafter, the three patients showed evidence of resorption of the construct. Again, this failure was attributed to the instability of the resorbable polymer following sustained dural pulsations that prevented the formation of stable bone. In summary, most of these studies show that the grafts appear to be successful in the short term, both clinically and radiologically; however, graft resorption is the main complication at the mid or long term.

### 3.2. Experimental Cell-Based Research

Preclinical in vivo data continue to be the most relevant evidence of the potential translation of any experimental cell-based strategy. During the last 2 decades, a great variety of cells combined with carriers or scaffolds have been tested in animal models of calvaria bone healing [60,61,62]. More recently, bioprinting has emerged as a technology with the potential to build “patient-specific” living constructs. “Cell-based but cell-free” bone tissue strategies are exploiting the paracrine control that biomolecules released from stem cells exert on host cells. To harness the potential of cell-based therapies, cell-based gene transfer approaches are also being explored.

#### 3.2.1. Research on Cell-Based Strategies

Although a few studies have explored the regenerative potential of terminally differentiated cells, e.g., osteoblasts [63,64], most research on cranial bone healing has been conducted with adult stem cells, mainly adipose- and bone marrow-derived stem cells, but also with muscle-, periodontal ligament-, neural crest-, cranial suture- and human dental pulp-derived stem cells, among others [65,66,67,68,69,70,71,72,73,74,75,76,77,78,79]. Collectively, most studies have reported the superior ability of adult stem cells to heal critical size calvarial defects compared with implantation of carriers or scaffolds lacking cells [65,66,73,75,77,78,79]. A study conducted on rabbit calvaria showed that the regeneration ability of bone marrow-derived stem cells is similar to that achieved by autologous bone [80]. Interestingly, recent data have shown that implantation of rat bone marrow-derived MSCs in cranial rat defects irradiated with a single dose of 20 Gy, equivalent to a conventional irradiation fraction protocol in human beings, improved bone neoformation compared with irradiated defects that did not receive cells [81]. Regarding the ability of the different sources of adult stem cells to heal calvarial defects, only a few studies comparing bone marrow-derived stem cells with other adult stem cells are available. Adipose-, human exfoliated deciduous tooth- and human dental pulp-derived stem cells led to outcomes comparable to bone marrow-derived cells, whereas periodontal ligament-derived stem cells led to poorer results [69,71,82]. Stem cells derived from placentome tissues, namely amniotic fluid, amniotic membrane and umbilical cord, are suitable allogenic cell sources for cranial tissue engineering [66,68,83,84,85]. Stem cells derived from term placentomes are excellent options for clinical use because they can be obtained using noninvasive methods and exhibit superior proliferation ability and lower immunogenicity than those derived from adult tissues. Human umbilical cord- and human bone marrow-derived MSCs seeded on CPCs showed similar regenerative efficacy, as shown after implantation in critical size defects of rat calvaria [68,83]. However, amniotic fluid-derived stem cells implanted in collagen scaffolds showed slightly higher osteogenic and angiogenic potential than dental pulp-derived stem cells [66].

Osteogenic and vasculogenic cells have been simultaneously co-transplanted into critical size cranial defects to stimulate de novo formation of blood vessels within the engineered bone [86]. To this end, both cell types were co-cultured in vitro prior to co-implantation. Encouraging results, in terms of enhanced bone healing compared with implantation of osteogenic MSCs only, were obtained by combining co-cultured endothelial progenitor cells with bone marrow- or adipose-derived MSCs in HA-based scaffolds [87,88]. Interestingly, Xu et al. [89] demonstrated that repair of critical size calvarial defects in rats can be promoted using osteogenic and endothelial cell sheets differentiated in vitro from bone marrow-derived MSCs, without the need for any scaffold providing structural support.

Compared with adult stem cells, embryonic stem cells (ESCs) are much more powerful tools for regenerative medicine applications. Whereas adult stem cells show limited proliferation capacity, especially when obtained from elderly individuals or from patients affected by systemic conditions, ESCs exhibit unlimited proliferation and self-renewal ability, providing a continuous supply of stem cells that can differentiate into a multitude of cell types from each of three germ layers with far superior efficiency than adult stem cells. Liu et al. [90] induced human ESCs to yield MSCs that were combined with CPC and implanted in critical size cranial defects of rats, resulting in enhanced bone healing compared with defects that only received cement. Interestingly, a comparison study of human ESCs and adult bone marrow-derived MSCs implanted in cranial defects of immunodeficient mice showed far greater bone repair in the animals that received the embryonic cells [91].

Like ESCs, induced pluripotent cells (iPSCs) are also capable of differentiating to a wide range of cell types. However, iPSCs surpass the ethical constraints for human ESC use because they can be obtained by reprogramming somatic cells. Moreover, given that they can be obtained from the patient’s own cells, they avoid the concern over immunogenic sequelae raised by allogenic stem cells while enabling the design of patient-specific cell therapies. Also like ESCs, iPSCs are prone to form tumors, a risk that can be circumvented prior to implantation by their induction into progenitor cells, such as MSCs, or fully differentiated cells [68,92,93,94]. A few studies have tested undifferentiated human iPSCs that were osteoinduced in vitro, combined with a carrier and then grafted into cranial rat defects. Bone healing was significantly higher in the defects that received osteoinduced human iPSCs than in defects that only received the carrier [68,92,93]. Interestingly, the quality of new bone was similar in the defects that received osteoinduced human iPSCs or human bone-marrow derived MSCs [68,92]. Given that major parts of the cranial skeleton originate from neural crest cells, neural crest-derived stem cells have been considered a potential cell source for cranial regeneration therapies. Although neural crest cells are very scarce in humans, they can be derived in large quantities from iPSCs [95]. Recently, iPSCs reprogrammed from human dermal fibroblasts were differentiated into neural crest cells and then into neural crest-derived mesenchymal progenitor cells [96]. These cells were seeded in a decellularized cranial allograft and implanted in defects created in the calvaria of non-obese diabetic/severe combined immunodeficient mice. Neural crest-derived mesenchymal progenitor cells enhanced cranial allograft integration into host bone to a higher extent than the allografts lacking cells or the allografts seeded with bone marrow-derived MSCs.

#### 3.2.2. Research on Cell-Based, Cell-Free Strategies

Engraftment and survival rates of transplanted MSCs are typically very low, remaining active only for a short time. Delivery of products secreted by these cells often results in regenerative outcomes similar to those observed after cell implantation. These observations have recently led to the shift of a paradigm centered on the progenitor function of MSCs to another based on the paracrine control exerted on host cells, which offers the possibility of bypassing the use of living cells for bone tissue engineering applications [97]. The use of the so-called “secretome,” which includes secreted structural and soluble molecules, provides for the opportunity to establish standardized “cell-based but cell-free” procedures. Cell-free strategies to deliver secreted products with regenerative activity explore the use of extracellular matrix, conditioned media and extracellular vesicles mainly derived from MSC cultures but also from other cell types.

The extracellular matrix behaves as a natural reservoir of a multitude of bioactive factors that are embedded in its structure. Chi et al. [98] prepared extracellular matrix suspensions obtained from cultures of rat bone marrow-derived MSCs that were incorporated into porous HA structures. Liu et al. [99] induced these cells toward the osteogenic lineage and employed a mild treatment with detergent to decellularize the extracellular matrix of the resulting cultures, obtaining a membrane that was used to wrap a collagen-based hydrogel containing graphene oxide nanosheets. Other authors incorporated the extracellular matrix from co-cultures of rat bone marrow-derived MSCs and a mouse osteoblastic cell line to a PCL membrane [100]. In these three studies, superior bone regeneration was observed after implantation of the various materials containing extracellular matrix in critical size defects of rats as compared with implantation of bare materials [98,99,100].

The conditioned media from cultured MSCs has also been assayed in rodent cranial defects. Katagiri et al. [101] soaked collagen sponges in conditioned medium from human bone marrow-derived MSCs. The sponges were implanted in the cranial defects of rats. Early bone healing was detected as soon as after 2 weeks, while the defects that received collagen soaked in a saline solution were covered by connective tissue. Using agarose as supportive material, the same group demonstrated that transplantation of human bone marrow-derived MSCs in rat calvarial defects leads to inferior results in terms of bone regeneration than media conditioned by these cells [102]. Delivery of atelocollagen containing conditioned media from human exfoliated deciduous tooth-derived MSCs in cranial defects of immunodeficient mice also resulted in greater formation of vascularized bone than delivery of the cells in the same carrier [103].

Extracellular vesicles, including exosomes and microvesicles, are lipid bilayer-delimited particles released from the cells that actively mediate intercellular communication. Among other biomolecules, they contain functional proteins, mRNAs, microRNAs and lipids, and they are very attractive candidates for cell-free applications in tissue engineering. Diomede et al. [104,105] isolated extracellular vesicles from human periodontal ligament- and gingival-derived MSCs, linked the vesicles to polyethylenimine and implanted them in rat cranial defects using collagen or PLA as carriers, respectively. The mineralization process as well as the development of an extensive vascular network was improved in animals receiving polyethylenimine-linked extracellular vesicles, compared with implantation with collagen or PLA. Exosomes, extracellular vesicles with submicron size diameters ranging from 30 to 200 nm, have also been tested in rat cranial defects [106,107,108,109,110]. Takeuchi et al. [106] implanted sponges of atelocollagen soaked with exosomes isolated from cultures of human bone marrow-derived MSCs. Significantly greater bone formation was observed in the defects implanted with exosomes compared with those that received atelocollagen only. Interestingly, the exosomes induced a notable accumulation of osteoblast-like cells and vascular endothelial cells and enhanced endogenous stem cell migration to the defect site. Exosomes isolated from human umbilical cord-derived MSCs also notably improved bone healing when implanted using hyaluronic acid-based hydrogels [107,108], whereas defects implanted with exosomes obtained from an immortalized human embryonic kidney cell line, which were used as a control, showed a poor repair response, similar to that of those defects that did not receive exosomes [108]. Exosomes from cultures of rat bone marrow-derived MSCs that were induced in vitro to the osteogenic lineage have also proven their ability to heal rat cranial defects after implantation in bioactive glasses [109]. Liang et al. [110] treated human bone marrow-derived MSCs with dimethyloxaloylglycine, a small molecule that induces the expression of hypoxia-inducible factor-1α (HIF-1α), which is a transcriptional activator that regulates coupling of angiogenic and osteogenic gene expression during bone development and repair, thereby with the potential to enhance new blood vessel formation in the engineered bone. Exosomes from cultures treated or not with dimethyloxaloylglycine were incorporated into HA scaffolds and implanted in rat cranial defects. Newly vascularized bone was markedly higher in defects implanted with exosomes from cells preconditioned with the small molecule compared with defects that received exosomes from untreated cultures.

#### 3.2.3. Research on Bioprinting

In recent decades, extensive research on tailored biomaterials that provide a suitable matrix for cell delivery that supports regeneration of the native, vascularized cranial bone structure has been conducted. To control the rate of bone regeneration, the chemical composition, architecture and mechanical properties of many biomaterials have been manipulated to improve their in vivo behavior [111,112,113,114]. A porous structure with interconnected pores has been found to be critical for cell ingrowth, but also for nutrient, oxygen and metabolic waste transport. Studies conducted with calcium phosphates-, PCL-, titanium- or collagen-based scaffolds indicate that pores smaller than 100 μm in diameter prevent oxygen and nutrient transportation into the scaffold inner and promote fibrous tissue formation, whereas pore sizes of at least 300–350 μm enhance osteogenesis and promote vascularization [111,112,113,114]. However, a high degree of open porosity can reduce the mechanical strength, especially in the case of biodegradable polymers, compromising the integrity of the material’s structure. Conventional methods used to prepare tissue engineered scaffolds often lack the ability to produce designs with precise structures. Although it remains common surgical practice to fill small cranial defects by hand modeling, this procedure might not be considered acceptable for areas of bare scalp, such as the frontal area of the skull. Computer-assisted 3-dimensional printing has emerged as an additive manufacturing technique to prepare personalized, shape-specific implants using digital data obtained by computed tomography or magnetic resonance imaging. Moreover, this bottom-up approach offers the opportunity to assemble individual components of the implant following a desired pattern to guide cell penetration and maturation toward functional tissue. By depositing a biocompatible material and viable cells, this technology evolved toward bioprinting, with the potential to construct “tailor-made” living constructs. Bioprinting has indeed shifted the paradigm of transplantation surgery, given that the need for donor organs could eventually be eliminated [115].

The formulation of bioinks with proper rheological properties is one of the main challenges in the development of functional constructs. Hydrogels are among the most commonly used base biomaterials for bioinks because their high water content facilitates cell entrapment [116]. Early bioprinting research was performed by simply adapting inkjet technology, in which individual droplets were used to generate a pattern. To prevent clogging and drop ejection, inkjet bioprinting employs low viscosity biomaterials, thus precluding the preparation of solid and large constructs for hard tissue repair. Other techniques, such as extrusion and laser-assisted methods, employ bioinks with increased viscosity, which sustain the transfer of cells at high densities. Extrusion, which uses a syringe and piston system to dispense the bioink through a microscale printhead to build a desired shape in a layer-by-layer fashion, has been successfully used to prepare constructs for craniofacial repair. To obtain a bioink with proper mechanical properties, Dubey et al. [117] entrapped dental pulp-derived MSCs in a synthetic hydrogel that incorporated amorphous magnesium particles of micrometric size. Constructs were successfully extruded using a cell-friendly print system and maintained structural integrity. The bioprinted constructs supported the osteogenic differentiation of the cells in the absence of chemical inducers, thereby highlighting their potential as bioinks for craniofacial repair. Chen et al. [118] envisaged a strategy in which autogenous, micron-scale particles of bone matrix were obtained by cryo-grinding of skull flaps and were mixed with PCL. The resulting slurry could be printed by extrusion to achieve a shape-specific implant. To test this approach, craniectomies were performed in rabbits to create critical size defects. The skull flaps were crushed, lyophilized, ground and finally used to print an implant with mechanical properties into the range displayed by trabecular bones. The implants were manually seeded with autologous bone marrow-derived MSCs and cultured for 2 weeks. One month after the craniectomy, a cranioplasty was performed by implanting the construct in the defect. Three months after, the constructs showed tight integration with the bone host tissue and generate vascularized mature bone, supporting the use of autogenous skull flaps as raw material in extrusion 3-dimensional printing. In the clinical scenario, bone matrix could be readily obtained after a cranial injury, although other anatomical sources (e.g., iliac crest or fibula) could also be used. Resolution of intractable intracranial hypertension [119] might provide for the time framework required for computer-aided design of implants and stem cell culturing, prior to cranioplasty.

Laser-assisted bioprinting (LAB) is based on the laser-induced forward-transfer effect, in which a laser pulse interacts with a sacrificial layer onto which the bioink film is placed, generating a cavitation-like bubble in the film. The expansion of the bubble propels the bioink to the substrate. The method allows droplet deposition of a fluidic phase at picoliter-level resolution [120]. LAB technology has been refined for direct in situ and in vivo bioprinting of biomaterials and constructs, therefore avoiding the need for ex vivo/in vitro culturing steps. In the field of cranial regeneration, the feasibility of this approach was initially tested by direct computer-assisted printing of nanohydroxyapatite (nHA) in calvarial defects created in mice [121]. Later, the same research group refined their technology to enhance bone regeneration of cranial defects by direct in situ printing of multipotent mouse bone marrow stromal precursor D1 cells [122]. After creating a calvaria defect, a layer of collagen and nHA was printed directly onto the dura mater of the mouse. Then, a cellularized ink, consisting of culture medium containing D1 cells that constitutively express a luciferase transgen, was printed on the layer, using either a “ring” or a “disk” pattern. Lastly, a second layer of collagen and nHA was printed over the cellularized printed ink. Six weeks later, bioluminescence assays revealed that printed cells remained viable, regardless of the pattern design used. Interestingly, the geometry of cell printing had a strong influence on bone regeneration. Constructs having cells printed as a “ring” only elicited marginal effects, whereas constructs with cells homogeneously printed as a “disk” resulted in enhanced bone regeneration. Given that the integration of a functional vascular network is still one of the major challenges of bone tissue engineering, LAB was assayed to print human umbilical vein endothelial cells (HUVECs) constitutively expressing a red fluorescent protein into bone defects created in the calvaria of immunodeficient mice [123]. A layer of collagen containing vascular endothelial growth factor (VEGF) and human stem cells from the apical papilla was directly deposited on the dura mater. HUVECs resuspended in culture medium were then bioprinted in situ on the layer, using various geometries that included “ring”, “disk” and “crossed circle” patterns. Then, a second layer of collagen containing VEGF and stem cells was overlaid. Cells printed with “ring” or “crossed circle” patterns led to organized microvascular networks. Increased vascularization correlated with enhanced bone regeneration compared with mice that did not receive endothelial cells or that received cells printed using a “disk” arrangement. These studies showed that stem and endothelial cells can be printed directly onto a bone defect using LAB, and that by using defined geometries, bone regeneration can be effectively modulated [122,123].

To address the limitations of the size and stability of constructs generated using extrusion and LAB technologies, a system called integrated tissue-organ printer was developed [124]. It employs multidispensing nozzles for delivering cell-laden hydrogels and synthetic biodegradable polymers, in a single construct. The system also prints a sacrificial hydrogel, Pluronic F-127, which is dissolved once the construct acquires enough rigidity and can maintain its shape. To assess cranial regeneration, human amniotic fluid-derived stem cells were included in a mixture of gelatin, fibrinogen, hyaluronic acid and glycerol and printed along with PCL containing tricalcium phosphate and the sacrificial hydrogel. To protect the cell-laden structures from external loads, the components were printed using a pattern that consisted of porous cell-laden hydrogel structures surrounded by a framework of PCL containing tricalcium phosphate in the outer layers and corners of each layer. After passaging through the nozzle system, thrombin was added, which resulted in rapid polymerization of fibrinogen, after which all the uncross-linked components (gelatin, HA, glycerol and Pluronic F-127) were washed out. Constructs in a circular shape were prepared and cultured for 10 days prior to implantation in rat calvarial bone defects. Five months after implantation, newly formed vascularized bone tissue was detected throughout the implants.

## 4. Cell-Based Gene Therapy for Cranial Bone Regeneration

Cell-based regional gene therapy, also known as ex vivo gene therapy, involves the implantation of genetically-modified cells to control the local production of a therapeutic transgene, and it has shown promise as a tissue engineering approach for bone regeneration. Prior to implantation of modified cells, this technology requires the collection of target cells from autologous or allogeneic sources, followed by a period of cell culture, expansion and gene transfer. Although this gene delivery method is rather cumbersome, the possibility of selecting well-characterized cell populations that can be further genetically modified with high efficiency is a major advantage over in vivo strategies. Moreover, ex vivo approaches in which target cells are transduced with viral vectors avoid the immunological risks associated with direct virus administration, and therefore are considered relatively safe. Numerous preclinical studies in animal models have demonstrated the ability of ex vivo regional gene therapy to safely and effectively heal bone defects, including critical size cranial defects [125,126]. Despite their promising properties, the usefulness of local application of recombinant growth factors, e.g., bone morphogenetic protein 2 (BMP-2), is compromised by their modest clinical efficacy and serious adverse effects due to the high doses of growth factor that need to be delivered at the defect site. Cell-based regional gene therapy, in combination with a variety of biomaterials, has emerged as an attractive alternative for growth factor delivery.

Transgenes encoding potent osteogenic BMP are the most extensively tested in ex vivo approaches for cranial bone regeneration. BMPs are a group of related proteins that, based on primary amino acid sequence homology, belong to the transformation growth factor-β superfamily [127]. Among the BMP members, recombinant human BMP-2 is a well-known inducer of bone regeneration, and it is used clinically in open tibial fracture healing and spinal fusion surgery. After genetic manipulation, various autologous or allogenic cell types have shown their ability to behave as BMP-2 protein-producing factories that enhance cranial bone regeneration. Regarding the research on suitable vectors to deliver BMP-2 transgenes, a comparative study tested rat bone marrow-derived MSCs transiently transfected with a cationic lipid or transduced with adenovirus or retrovirus [128]. Genetically modified cells were seeded in a titanium mesh and implanted in critical size calvaria defects of rats. The adenoviral vector led to a slight but statistically significant increase in bone formation compared with non-modified, transiently transfected or retrovirally transduced cells. A subsequent study tested rat bone marrow-derived MSCs that were transiently transfected using a cationic polymer with a plasmid encoding human BMP-2, then seeded in a gelatin scaffold and implanted in rat critical size defects [129]. There were no significant differences in the new bone area of defects that received unmodified or modified cells. In these two studies [128,129], the lack of enhanced bone healing efficiency could be attributed to a deficient experimental design that led to insufficient production of BMP-2 in transfected and retrovirally transduced cells. In fact, enhanced bone healing in the skull defects of rats could be detected after implantation of rat bone marrow-derived MSCs that had been transiently transfected, using a lipid-polymer mixture, with a plasmid carrying a human BMP-2 gene [130].

The tetrameric cell penetrating peptide Tat has been combined with adenovirus encoding human BMP-2 as a strategy to increase transduction of human bone marrow-derived MSCs. It resulted in far greater new bone formation in rat critical size defects than in those that received cells transduced only with adenovirus [131]. Improved transduction of human adipose-derived MSCs using the same methodology also led to much more effective healing of rat cranial defects than that achieved by non-transduced cells [132]. Sun et al. [133] developed a single-step visible light photo-crosslinking method of fabrication of an injectable, biodegradable gelatin scaffold including recombinant adeno-associated adenovirus encoding human BMP-2 that was able to transduce in situ human bone marrow-derived MSCs encapsulated within the scaffold. New bone formation was detected in cranial defects of severe immunodeficient mice treated using this methodology, which does not require ex vivo gene transfer. Injectable materials avoid using preformed scaffolds, the inner space of which is difficult to colonize by genetically modified cells and are especially suitable for minimally invasive surgical procedures. He et al. [134] developed an injectable system that employed a paste of nano-scale calcium sulphate and alginate. Rat bone marrow-derived MSCs were transduced with an adenovirus encoding human BMP-2, mixed with the paste and injected into the critical size cranial defects of rats. Nearly complete closure of the bony defects was detected in these animals, whereas the cells transduced with a reporter gene only induced partial bone healing.

Particularly impressive were the results obtained by Chang et al. [135] with bone marrow-derived MSCs isolated from miniature pigs. The cells were expanded and transduced 1 week before craniectomy with an adenoviral vector encoding human BMP-2. Autologous, genetically modified cells were mixed with collagen type I and implanted in skull defects with an average area of approximately 7 cm^2^. Near-complete, full-thickness cranium defect repair was detected after cranioplasty using BMP-2-transduced cells, whereas the defects that received unmodified cells lacked consistent bone formation at the center of the defects. The stiffness of the engineered bone was indistinguishable from that of normal bone. A subsequent study from the same group [136], in which BMP-2-transduced autologous cells were seeded on an osteoconductive scaffold composed of gelatin and TCP, also showed enhanced bone formation in circular 4-cm diameter skull defects compared with non-transduced cells. Encouraging data were also obtained in the same large animal model after filling critical size defects with a mixture of polyethylene glycol and biphasic calcium phosphate containing human fetal osteoblasts transfected with a BMP-2 gene [137,138].

Differentiated cells have also been explored in preclinical gene therapies to enhance cranial bone regeneration through production of exogenous BMP-2. Lee et al. [139] transduced muscle-derived cells obtained from male immunocompetent mice with a replication-deficient adenovirus encoding human BMP-2. The genetically modified cells were seeded in collagen sponges and implanted in critical size defects created in the skull of severe combined immunodeficiency female mice. The results of this pioneering work proved that intralesional delivery of muscle cells expressing the BMP-2 transgene greatly enhanced bone regeneration, whereas non-transduced cells led to limited healing of the defects. The results also showed that Y chromosome-positive transduced cells located within the newly formed bone of the female mice stained for osteocalcin, indicating that they not only participate as cell factories for BMP-2 secretion but also contribute to repairing the defect by differentiating in vivo toward the osteogenic lineage. Liu et al. [140] tested an expedited, regional gene transfer strategy for skull healing in which muscle grafts are obtained, transduced and implanted within the framework of a single surgery, therefore avoiding time-consuming ex vivo culturing procedures. Autologous rat muscle biopsies were transduced intraoperatively with adenovirus carrying a human BMP-2 gene and, while the biopsies were transduced, a parietal cranial defect was created in the same animal. Implantation of these transduced grafts resulted in the deposition of more than twice the new bone as occurred in defects filled with non-transduced grafts or grafts transduced with a reporter gene. Fibroblasts can be easily isolated from skin, revealing themselves as potential gene delivery carriers for ex vivo gene therapy. Syngeneic and autologous dermal fibroblasts were transduced with a retrovirus carrying a human BMP-2 gene and then implanted in gelatin sponges in rat cranial defects [141]. The osteoinductive capability of the exogenous BMP-2 released by syngrafts and autografts enhanced bone osteogenesis, whereas the defects that received non-modified fibroblasts mainly developed fibrous tissue.

BMP-7 is a member of the BMP family with strong osteoinductive activity. In 2001, the recombinant version of human BMP-7 received a limited US Food and Drug Administration approval for the treatment of recalcitrant tibial non-unions. A large, prospective, randomized-controlled, multicenter clinical trial did not show that BMP-7 treatment is truly non-inferior to iliac crest autograft and, consequently, there have been no products on the market based on this protein since 2009. However, research on ex vivo regional gene therapy to overexpress BMP-7 in cranial defects has yielded interesting data. Rat dermal fibroblasts that were transduced with an adenoviral vector carrying a BMP-7 gene led to complete repair of rat cranial defects [142]. In addition to secreting biologically active BMP-7 in vivo, the transduced, non-osteogenic fibroblasts also differentiated into bone-forming cells. Interestingly, this ex vivo gene therapy approach successfully enhanced healing of rat cranial defects that had been severely compromised with a single 12-Gy radiation dose delivered 2 weeks after the cranioplasty [143].

BMP-4 is a major regulator of the development of axial and craniofacial structures of the skeleton. The potential of a human BMP4 transgene to regenerate skull defects using an ex vivo gene therapy approach was tested in retrovirally transduced rat bone marrow stromal cells that were included in a gelatin matrix and implanted in rat calvaria defects. The defects completely filled with new bone, while limited bone formation occurred in defects that received non-transduced cells [144]. Rat muscle-derived stem cells and primary muscle-derived cells transduced with a retroviral vector encoding human BMP-4 were able to heal critical size defects of mice and rats [145,146]. A comparison study between the osteoinductive ability of transduced rat muscle-derived stem cells and primary muscle-derived cells showed that, surprisingly, healing of rat calvaria defects was superior when BMP-4 was delivered by primary cells [146]. A study conducted with murine muscle-derived stem cells transduced with a retrovirus encoding human BMP-4 revealed that the cells implanted in the cranial defects of mice not only contributed directly to the regeneration of the bone structure via their differentiation into chondrocytes, osteoblasts and osteocytes, but also through paracrine effects that led to faster inflammation resolution and enhanced angiogenesis [147]. Murine autologous bone marrow- and adipose-derived MSCs transduced with an adenovirus carrying a BMP-4 gene also behaved as suitable cellular vehicles that enhanced bone healing of rabbit skull defects to a similar extent [148].

The therapeutic potential of BMP-9 for promoting cranial bone growth has been tested in immortalized mouse calvarial mesenchymal progenitor cells that were transduced with an adenovirus carrying a human BMP-9 gene and then implanted in athymic mice [149]. As supportive material, a thermoresponsive biomacromolecule was used that undergoes a liquid-to-solid phase change at physiological temperature, molding to the shape of the defect. The delivery of transduced cells promoted mature bone formation, effectively repairing the cranial defect. Clustered regularly interspaced short palindromic repeats/associated nuclease Cas9 (CRISPR-Cas9) activation has recently been used to induce endogenous BMP-9 in immortalized mouse bone-marrow-derived MSCs transduced with lentiviral vectors [150]. Two weeks after generating a cranial defect, BMP-9 overexpressing cells were directly injected in the lesion using the newly formed connective tissue as a natural scaffold to retain the cells in the defect. This approach, which addresses a common clinical situation in which early cranioplasty cannot be performed, led to increased bone healing compared with defects injected with cells that do not overexpress BMP-9.

Noggin is a secreted, high-affinity BMP antagonist protein, which functions by binding directly to several BMPs, including BMP-2, BMP-4 and BMP-7, thereby blocking their interaction with specific cell surface receptors [151]. Levi et al. [152] hypothesized that the osteogenic potential of stem cells could be greatly enhanced by suppressing their noggin production. To test this, human adipose-derived MSCs transduced with a lentiviral vector carrying a noggin short hairpin RNA (shRNA) were implanted in critical size defects created in nude mice. Noggin knockdown led to increased BMP-2 activity within the cranial defects, which accelerated the repair of the lesion compared with defects that received cells transduced with a vector carrying a control shRNA. A recent study explored simultaneous noggin suppression and BMP-2 overexpression in rat adipose-derived stem cells [153]. Cells were co-transduced with baculovirus vectors carrying a BMP-2 gene and a CRISPR interference system to target endogenous noggin and then implanted in rat cranial defects using gelatin as carrier. Bone healing driven by BMP-2 overexpression was far superior in the noggin-suppressed cells than in the cells in which noggin expression had not been altered.

In addition to BMP proteins and antagonists, secretion of other growth factors and ligands has been manipulated by means of gene transfer to enhance cranial bone regeneration. To overexpress VEGF, one of the most potent inducers of angiogenesis, human adipose-derived MSCs were transfected with a plasmid encoding the human VEGF gene [154]. Bone healing and neovascularization of the cranial defects of immunosuppressed rats filled with a scaffold containing bone marrow-derived cells and a small proportion of transfected cells were significantly greater than in the defects that only received bone marrow cells. It was noted that, due to excessive secretion of VEGF, induced osteogenesis was lower when high proportions of transfected cells to bone marrow cells were used. Basic fibroblast growth factor (bFGF) is a protein that participates actively in osteogenesis and angiogenesis during skeletal healing. bFGF gene transfer to bone marrow-derived MSCs that were implanted in rat calvaria defects accelerated their vascularization [155] and bone regeneration [156]. By triggering canonical Wnt signaling, Wnt10b promotes osteogenic differentiation and represses adipogenic differentiation. Increased blood vessel growth and accelerated bone healing was observed in rat calvaria defects after implantation of human umbilical cord MSCs transduced with a lentivirus that encodes rat Wnt10b [157].

Diffusion of secreted transgenic proteins from cell constructs implanted in the cranial lesion can result in uncontrolled osteogenic and/or angiogenic responses. As an alternative, regional gene therapy involves the use of bone-specific transcription factors to drive differentiation of stem cells toward the osteoblastic lineage. Osteogenesis depends upon the activity of at least 2 transcription factors, Runx2 and osterix. Runx2 is an osteoblast-specific transcriptional activator that controls osteoblast development and maturation from MSCs. Mouse bone marrow-derived cells transduced with an adenovirus encoding Runx2 were adsorbed in gelatin sponges and implanted in craniotomy defects performed in syngenic mice [158]. Defects that received cells overexpressing Runx2 were completely repaired, whereas those that received cells overexpressing a reporter gene showed partial healing. The effects of Runx2 overexpression on cranial repair appear to be strongly dependent on the target cell population. Thus, negligible repair of craniotomy defects was observed after implantation of skin fibroblasts transduced with an adenovirus encoding Runx2, whereas fibroblasts genetically modified with an adenoviral vector to overexpress BMP-2 demonstrated effective healing [159]. Osterix is another essential osteoblast-specific transcription factor that acts downstream of Runx2 to induce differentiation of pre-osteoblasts into fully functional osteoblasts that, in mice, is indispensable for bone formation. To test whether overexpression of this transcriptional activator could enhance bone healing, bone marrow-derived MSCs were transduced with a retrovirus encoding osterix [160]. Using collagen sponges as carriers, the transduced cells were implanted in calvaria defects of mice. Implantation of osterix-overexpressing cells resulted in the nearly complete healing of skull defects, the amounts of newly formed bone being 5 times higher than in the defects that received cells transduced with an empty vector.

Rat bone marrow-derived MSCs transduced with a lentivirus encoding HIF-1α increased bone volume and mineral density as well as blood vessel number and area after implantation in rat cranial defects compared with defects that were implanted with cells transduced with a lentivirus carrying a reporter gene [161,162,163]. Cells engineered to overexpress constitutively active mutants of the transcription factor instead of the wild-type protein further enhanced osteogenic and angiogenic responses in the cranial lesions [161,162,163]. Special AT-rich sequence-binding protein 2 (SATB2) is a nuclear matrix protein that regulates many genes’ activities during osteoblast differentiation and is thereby involved in craniofacial development. To investigate whether this protein could contribute to bone repair, mouse iPSCs were retrovirally transduced to overexpress mouse SATB2 and cultured for two weeks in osteogenic medium [164]. The transduced iPSC were seeded in silk fibroin scaffolds and then implanted in critical size cranial defects of nude mice. The cranial defects filled with newly formed bone tissue and showed nearly complete osseous closure while the defects implanted with iPSC transduced with an empty vector led to a substantially lower amount of irregularly arranged bone tissue. Histone lysine demethylase PHF8 is an epigenetic modifier that regulates osteogenic differentiation of bone marrow-derived MSCs by activating SATB2 transcription [165]. Mouse bone marrow-derived MSCs overexpressing PHF8 were seeded in silk fibroin scaffolds and implanted in critical size cranial defects of immunocompetent mice. The implanted cells underwent osteogenic differentiation and led to large new bone formation within the defects [165].

Osteogenesis can be dramatically enhanced using regional gene therapy to express specific combinations of interacting regenerative molecules. For example, more effective healing was observed in cranial defects of mice implanted with a gelatin scaffold containing mouse fibroblasts transduced with adenovirus carrying BMP-2 and BMP-7 genes than in defects that received cells transduced with adenovirus carrying a BMP-2 or a BMP-7 gene [166]. To study the interactions between BMP-2 and VEGF, mouse muscle-derived stem cells were retrovirally transduced to overexpress human BMP-2 and VEGF [167]. Critical size calvaria defects of mice were implanted with collagen scaffolds impregnated with BMP-2 or with BMP-2- and VEGF-expressing cells. Exogenous production of VEGF improved BMP2-induced bone healing by facilitating angiogenesis, which accelerated cartilage resorption and enhanced mineralized bone formation. Similar results were obtained in experiments in which mouse muscle-derived stem cells were retrovirally transduced to overexpress human BMP-4 and VEGF [168]. The interaction between these growth factors was critically dependent on their proportions, given that decreased synergism was detected with higher ratios of VEGF-expressing cells than BMP-2- or BMP-4-expressing cells. Furthermore, sustained production of BMP-2 and VEGF by baculovirus-transduced rabbit bone marrow-derived MSCs that were implanted in rabbit skull defects significantly improved bone healing [169]. Cell delivery of chemically modified mRNAs has recently gained interest as a non-integrating, non-viral approach that could be exploited in bone tissue engineering [170]. Rat bone marrow stem cells were engineered with chemically modified mRNAs encoding human BMP-2 and VEGF, which increased cell secretion of both growth factors. Cells were implanted in rat cranial defects, using a collagen fiber matrix as a supportive material. The cells treated with modified mRNAs encoding BMP-2 and VEGF led to bone formation superior to the non-treated cells or to the cells that were treated with mRNAs encoding BMP-2 or VEGF. Stromal cell-derived factor 1 (SDF-1) is a chemokine that can trigger the migration of bone marrow-derived MSCs, playing a key role in their homing to the fracture site. Lo et al. [171] developed a baculovirus vector to co-deliver BMP-2 and SDF-1 genes to rat adipose-derived MSCs. Smad and ERK1/2 pathways were synergistically activated in the engineered cells, which after implantation using gelatin scaffolds in calvaria rat defects outperformed to a great extent the bone repair achieved by overexpression of BMP-2 or SDF-1 alone. Sox9 and PPAR-γ are the master transcription factors that govern chondrogenesis and adipogenesis, respectively. Truong et al. [172] designed a CRISPR-based system to simultaneously activate Sox9 and inhibit PPAR-γ. The components of the system were packaged in a baculovirus vector, which was used to transduce rat bone-marrow derived MSCs. Engineered cells, seeded in gelatin scaffolds and implanted in rat calvaria defects, remarkably ameliorated bone growth compared with non-transduced cells. FOXC2 is a transcription factor that induces the non-canonical Wnt signaling pathway, contributing to bone marrow osteogenesis as effectively as the canonical Wnt pathway induced by Wnt10b [173]. Rat bone marrow-derived MSCs transduced with a baculovirus encoding a CRISPR system to co-activate endogenous Wnt10b and Foxc2 greatly improved bone healing after implantation into critical size calvaria defects of rats [173].

The potential of proteins that participate in the regulation of osteoblast differentiation, other than secreted growth factors and lineage-specific transcription factors, has been explored in ex vivo gene therapies for cranial repair. Tested proteins include α5 integrin, which activates signaling pathways of human bone marrow-derived MSCs, resulting in increased Runx2 expression that promotes osteogenic differentiation [174]; ubiquitin-specific protease 53 that promotes osteogenesis in human bone marrow-derived MSCs by activating the canonical Wnt signaling pathway [175]; and pannexin 3, a glycoprotein essential for hard tissue development, which forms hemichannels and facilitates the passage of ions and small molecules from the intracellular space to the extracellular microenvironment [176]. Overexpression of α5 integrin, ubiquitin-specific protease 53 or pannexin 3 in human MSCs significantly enhanced bone healing of cranial defects created in rodents compared with cells that had not been genetically modified [174,175,176].

In addition to specific signaling pathways and transcription factors, osteogenesis is tightly controlled by microRNAs (miRNAs), small non-coding RNAs that behave as key post-transcriptional regulators of gene expression through induction of either translational repression or cleavage of targeted mRNAs. Some studies have recently engineered MSCs to overexpress miRNAs that modulate their differentiation toward the osteoblastic lineage. miR-135 negatively regulates the levels of the homeobox Hoxa2, a protein that restricts bone mineralization in the calvaria during craniofacial development. Xie et al. [177] overexpressed miR-135 in rat adipose-derived MSCs. Implantation of cells overexpressing miR-135 in cranial defects of rats significantly enhanced bone healing. Moncal et al. [178] explored the overexpression of miR-148b, a miRNA that stimulates osteogenesis by targeting noggin mRNA [179]. Rat bone marrow-derived MSCs were transfected with miR-148b using a silver nanoparticle system, encapsulated in collagen and seeded in 3D-printed scaffolds that were implanted in critical size calvaria defects of rats. Untransfected cells led to immature bone formation, whereas miR-148b-transfected cells nearly filled the entire defects with dense, mature bone tissue. More potent effects, in terms of bone healing of cranial defects generated in nude mice, were detected after implantation of human adipose-derived MSCs that were transduced with a baculovirus vector to co-express BMP-2 and miR-148b than with cells engineered to only express BMP-2 or miR-148b [179]. Implantation of lentivirally transduced bone marrow-derived MSCs overexpressing miR-26a [180] or miR-129-5p [181] also accelerated healing of mouse cranial defects.

### Cell-Based Regulatable Gene Therapy for Cranial Bone Regeneration

All the gene expression systems described thus far employ constitutively active promoters, which are unregulated and can produce excessive amounts of transgenic products, leading to adverse effects like those caused by delivery of recombinant proteins. Ideally, the expression of the therapeutic transgenes should be temporally coupled with the progression of healing to provide for the best control of the cranial regeneration process. However, unregulated promoters cannot limit transgene expression to the optimal therapeutic window. Currently available pharmacologically activated gene expression systems can regulate the level and duration of transgene expression, thus increasing the safety and efficacy of gene therapy.

Temporal regulation can be achieved through the use of systems that function as on/off gene switches. They generally comprise a chimeric transcription factor that is activated or inactivated by a small-molecule ligand and a promoter linked to the transgene to be regulated, which is activated or silenced by the transcription factor. The Tet-On system employs transactivators based on a mutant of the tetracycline repressor protein that in the presence of tetracycline or derivatives such as doxycycline bind to a responsive promoter. Peng et al. [182] transduced muscle-derived stem cells with a retroviral vector carrying a BMP4 gene under the control of a Tet-On system. Gelatin disks were impregnated with transduced cells and implanted in mouse cranial defects. The animals were administered with doxycycline for 10 days after surgery, which led to complete bone healing. However, a control group of mice not treated with doxycycline showed residual mineralized bone formation, probably due to basal affinity of the transactivator for its responsive promoter in the absence of inducer, which caused significant BMP-4 production. To overcome undesired bone regeneration, the authors co-implanted cells transduced with a retrovirus that controls BMP-4 production employing the Tet-On system and a small proportion of cells transduced with retrovirus that drive noggin expression using a constitutive, unregulated promoter. Introducing noggin production into the system not only blocked the osteogenic activity of the basal amount of BMP-4 secreted in the absence of the inducer, but also prevented bone overgrowth in the presence of inducer, resulting in the formation of new bone similar to the original tissue. Chen et al. [183] used a Tet-Off system based on the tetracycline repressor protein that in the absence of tetracycline or derivatives binds to a responsive promoter whereas in its presence dissociates from the promoter, silencing transgene expression. Using an adeno-associated viral vector, the system was used to control the transgenic expression of bFGF in rat bone marrow-derived MSCs. Transduced cells were implanted in the cranial defects of rats that were administered with doxycycline to repress bFGF production. Only minimal new bone formation was observed in the skull defects. The control group that was not treated with doxycycline increased bFGF production, which greatly enhanced bone regeneration and angiogenesis.

Dimerizer-activated gene expression systems use a synthetic transactivator in which DNA binding and activation domains are distributed in 2 different non-interacting proteins, each of which has affinity to a dimerizer molecule (e.g., rapamycin, a macrolide approved for human use, or their non-immunosuppressant analogues), but not to the other protein. Only after a dimerizer capable of joining both molecules is added can the separate components form a functional transcription factor. Koh et al. [184] explored the use of a rapamycin-regulated system for controlling bone regeneration. To this end, embryonic mouse fibroblasts were transduced with retrovirus carrying a BMP-2 gene under the control of the system. Transduced fibroblasts were implanted in a cranial lesion generated in mice. Administration of rapamycin induced significant healing of the defects, which was not observed in the absence of rapamycin treatment. A group of mice were implanted with fibroblasts transduced with an adenovirus carrying a constitutively-expressed BMP-2 gene. This unregulated system led to overgrowth of new bone, which was highly irregular and discontinuous with host bone while bone formation induced by the rapamycin-induced system was uniform across the lesion area and fully integrated with the host.

During bone development and healing, cellular gene expression is tightly controlled in space [185,186]. Creating well-defined physiologic patterns of regenerative molecules in implanted cell constructs, however, remains as a substantial challenge that has motivated the development of transcriptional strategies for controlling spatial presentation of bioactive factors. A strategy to achieve spatial control of gene expression relies on the use of targetable promoters that can be externally activated by directed physical forces. Heat shock protein (HSP) gene promoters can restrict the expression of transgenes to desired target regions by localized heating using, e.g., focused ultrasounds or infrared irradiation [187]. Heat induction of HSP promoters occurs in virtually every cell type, being promoter activity determined, within certain limits, by the activating heat dose that is a function of both temperature and length of exposure. Thus, a therapeutic gene could be expressed at a specific location of any target tissue at the level that produces optimal results. HSP promoters are silenced within a few hours of the activating heat treatment, adding an element of therapeutic safety but also limiting their usefulness because they might not direct transgene activity over extended periods of time. Of even more concern is the involuntary activation of HSP promoter-controlled therapeutic genes caused by a rise in body temperature that could result in unintended transgenic expression. To avoid these problems, we developed a new generation of regulatory circuits that combine an HSP70B promoter and a small molecule-dependent transactivator that can provide for both spatial and temporal control of transgene activity [188]. A particular system employs a heat-activated HSP70B promoter to drive the expression of a chimeric transactivator that in the presence of rapamycin or its non-immunosuppressive analogs acquires transcriptional competence and controls the expression of a therapeutic transgene [189]. A cell line was derived from the mouse embryo cell line C3H/T101/2 that stably harbored a luciferase gene under the control of the gene switch. The engineered cells were distributed in a fibrin hydrogel and subcutaneously implanted in the backs of syngenic mice. Animals administered with dimerizer were subjected to localized heating by partial immersion in a water bath, which resulted in >250 fold induction of reporter activity. The switch was only activated by heat in the presence of rapamycin or rapalog AP21967, maintained high-level reporter expression for several days after heat activation and was silenced by removal of ligand. A further cell line was generated that stably integrated a VEGF gene under control of the heat-activated, dimerizer-dependent gene switch. The cells were tested as described above. Skin and muscle responses to released VEGF from activated implants included strong staining of Von Willebrand factor, indicating vascular reorganization. Gluteal muscles underlying activated implants were hyperemic, showed mononuclear cell infiltrates and increased expression of CD31. The stringent requirement for both heat and dimerizer stimuli prevented the production of VEGF in non-heated locations as well as in mice that were only subjected to heat treatment or only administered with rapamycin. In summary, these data indicate that the heat-activated, ligand-dependent switches provided control of both location and duration of therapeutic transgene expression.

**Figure 3:**
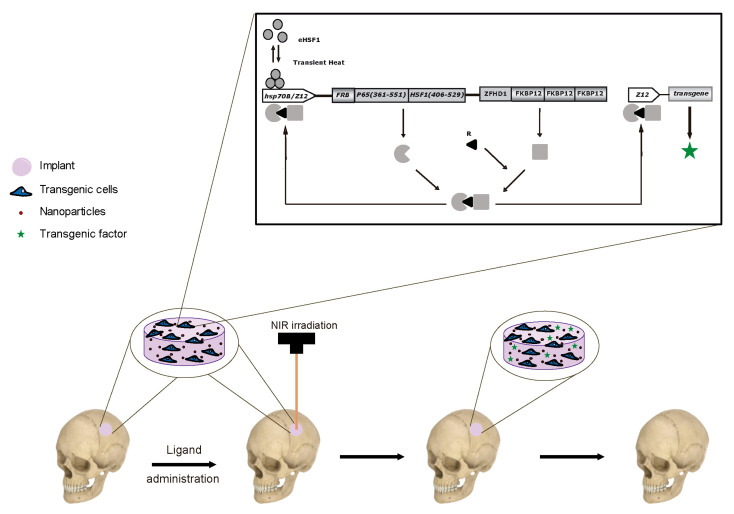
Cranioplasty conducted using NIR-responsive implants. A scaffold containing genetically modified cells that harbor a heat-activated and dimerizer-dependent gene expression system to control the expression of a transgene is implanted in a cranial lesion. The gene expression system is composed of a bi-cistronic gene encoding the 2 component proteins of a dimerizer-regulated transactivator and is expressed under the control of promoter cassette hsp70B/Z12, which responds to activated endogenous heat shock factor 1 (eHSF1) as well as the dimerizer-activated transactivator. Transactivator-responsive promoter Z12 controls the linked transgene. R: rapamycin. For additional details on the expression system, see [187]. After administration of a dimerizer, the implantation area is NIR-irradiated, resulting in local activation of transgene expression that stimulates bone healing. The figure includes an image retrieved from https://free3d.com/es/modelo-3d/caucasoid-male-skull-4837.html, accessed on 30 August 2021.

Recent advances in nanotechnology have provided for noninvasive means of heating tissues. Near infrared (NIR) light from the 650–900 nm range is minimally absorbed by skin and underlying tissues, which leads to deep tissue penetration and minimal tissue damage. Various nanostructures and probes can absorb energy from irradiating NIR light and convert it into heat to increase the temperature of the surrounding environment [190]. Photothermal treatments driven by plasmonic nanomaterials such as carbon nanohorns and hollow gold nanoparticles (HGNP) have proven useful for local activation of transgenes controlled by thermosensitive promoters [191,192]. Our group has explored the ability of fibrin-based hydrogels loaded with HGNP to transduce photon energy into heat [193,194]. We used these biocompatible composites as scaffolds for harboring genetically modified murine multipotent stem C3H/T101/2 cells that contain the heat-activated and dimerizer-dependent gene expression system to control the expression of a luciferase gene. Noninvasive NIR irradiation made it possible to generate well-defined patterns of luciferase activity in scaffolds implanted subcutaneously in syngenic mice administered with rapamycin [193]. Interestingly, this approach was useful for defining in vivo expression patterns of VEGF. More recently, we prepared NIR-responsive fibrin-based cell constructs to regulate BMP-2 secretion from genetically modified cells. In vitro experiments indicated that BMP-2 was released from the hydrogels only upon photoinduced mild hyperthermia in the presence of dimerizer, but not after NIR irradiation alone or dimerizer treatment alone. Moreover, the growth factor released from activated constructs showed strong autocrine and paracrine activity. To test the effectiveness of this approach in regulating the secretion of BMP-2 in vivo, NIR-responsive hydrogels were implanted in critical size defects generated in the parietal bone of mice that were administered rapamycin and NIR-irradiated 1 and 8 days after implantation. NIR irradiation of the implanted area significantly stimulated BMP-2 production in the hydrogels from the animals treated with rapamycin, resulting in bone regeneration from the edge of osteotomy, whereas no significant BMP-2 production could be detected in implants from the non-irradiated animals, which only showed a thin layer of fibrous tissue bridging the defect [195]. Thus, deliberate patterning of transgene expression in NIR-responsive hydrogels implanted in a bone defect can be a useful approach for achieving spatiotemporal control of regenerative growth factors, thereby mimicking endogenous bone repair mechanisms. This technology (Figure 3), which can be adapted to a variety of photothermal materials, can be further optimized for cranioplasty procedures. Autologous cells, expanded and genetically modified ex vivo, would be implanted in the lesion using a photothermal carrier. To avoid cumbersome ex-vivo manipulation steps, cells could be obtained and genetically modified intraoperatively. Expression of a transgene promoting bone healing could be achieved in the cranioplasty area by NIR irradiation after administration of rapamycin. By employing another available heat-activated and ligand-dependent gene switch that employs a transactivator regulated by mifepristone or ulipristal [188,196], a second transgene could be expressed by a second round of activation in a different timeframe. Currently available technologies allow adapting NIR energy deposition to the size and shape of the target, achieving well-defined transgenic patterns that faithfully match the irradiated motif. Conditions that can be modulated to optimize laser application to the implanted area include power density control, irradiation time and dispersion of NIR energy through the use of light distributors, diffusers or laser arrays, which can also define the geometry of the irradiated areas. Finally, easy handling and high safety standards of NIR lasers could facilitate the translation of NIR-responsive, cell-based gene therapy technology.

## 5. Concluding Remarks and Future Directions

Bone tissue engineering offers the promising alternative of generating constructs, composed of biomaterials including cells and/or growth factors that can enhance the outcome of reconstructive treatments. Thus far, the clinical applications of stem cells in cranial bone reconstructive surgery have been limited to small case series with varying outcomes. Although data collected from these studies have shown the safety of cell-based therapy, the great variability of indications, defect sites and sizes, as well as stem cell sources and biomaterials makes it difficult to draw solid conclusions on stem cell efficacy in cranial tissue engineering. In fact, the poor understanding of the therapeutic mechanism of action of the implanted cells, as well as the lack of technologies to control their fate determination, are the main obstacles for the clinical translation of cell-based therapies. Development of methods for identifying, isolating and expanding large quantities of specific populations of functional cells will result in more efficacious therapeutic products. Nonetheless, stem cell-based therapies have already emerged for the treatment of cranial defects, and it is expected that future clinical trials will succeed in showing their efficacy.

Preclinical data obtained using animal models are, however, much more encouraging. Many cell types, in combination with biomaterials, some designed to stimulate cellular responses, have proven their ability to accelerate bone healing after implantation in defects created in various animal models. Data obtained using extracellular matrix, conditioned media and exosomes strongly support the hypothesis that cranial bone regeneration can be achieved through mobilization of endogenous cells using “cell-based but cell-free” approaches. Nevertheless, prior to manufacturing therapeutic products with standardized benefits, more fundamental knowledge on these released systems is needed to identify those key components that play a specific role in the bone healing cascade. Lastly, many studies have convincingly shown that bone regeneration of cranial defects could be enhanced by genetic manipulation of cells from various sources. The overexpression of genes encoding secreted growth factors and ligands, transcription factors and signaling molecules, alone and in combination, have been tested in cell-based approaches. More recently, the potential of miRNA overexpression has started to be explored. Given that viral vectors with improved safety profiles and non-viral vectors with improved gene transfer efficiencies are being developed, bone tissue engineering employing ex vivo regional gene therapy could become a clinical reality within the next few years. Moreover, recently developed transcriptional strategies could provide for tight spatial and temporal control of transgenic expression. Cell-based therapies will undoubtedly benefit from bioprinting techniques. With further development, bioprinting could produce clinically useful computer-assisted, patient-specific constructs that incorporate controlled patterns of multiple cell types, transgenic or not, to recapitulate the native structure and function of vascularized bone tissue. Particularly exciting are the data obtained by means of in vivo bioprinting, which pave the way to the intraoperatively application of this technology.

Aging, systemic diseases, radiation and infection are factors that critically affect healing of critical sized bone defects. While most of the preclinical studies have been conducted with healthy young animals, research on the influence of those conditions in cranial repair induced by cell therapies is scarce. In most cases, materials harboring cells are implanted immediately after the critical defect is generated, an experimental approach that does not mimic the real clinical situations, e.g., cranioplasties resulting from decompressive craniectomies, which are only performed after resolution of the cerebral edema. The comprehensive consideration of these factors might accelerate the regulatory approval and clinical translation of cell-based products for cranial repair.

Given the complexity of cell bioprocessing, manufacturing of cell-based therapies involves a high level of structural and economic resources. Research advancements on the field of cranial bone regeneration will be fruitless if the overall cell production pipelines are not cost-effective. In this regard, substantial efforts have been recently made by implementing automated process steps that reduce the cell production costs. Despite the challenges, cell-based technologies show unprecedented levels of therapeutic potential and are expected to radically change the landscape of cranial bone reconstruction in the near future.
